# Time to Spread Your Wings: A Review of the Avian Ancient DNA Field

**DOI:** 10.3390/genes8070184

**Published:** 2017-07-18

**Authors:** Alicia Grealy, Nicolas J. Rawlence, Michael Bunce

**Affiliations:** 1 Trace and Environmental DNA (TrEnD) Laboratory, Department of Environment and Agriculture, Curtin University, Bentley, Perth, WA 6102, Australia; michael.bunce@curtin.edu.au; 2 Otago Palaeogenetics Laboratory, Department of Zoology, University of Otago, Dunedin 9010, New Zealand; nic.rawlence@otago.ac.nz

**Keywords:** aDNA, ancient DNA, archaeology, aves, avian, bird, conservation, ornithology, palaeontology

## Abstract

Ancient DNA (aDNA) has the ability to inform the evolutionary history of both extant and extinct taxa; however, the use of aDNA in the study of avian evolution is lacking in comparison to other vertebrates, despite birds being one of the most species-rich vertebrate classes. Here, we review the field of “avian ancient DNA” by summarising the past three decades of literature on this topic. Most studies over this time have used avian aDNA to reconstruct phylogenetic relationships and clarify taxonomy based on the sequencing of a few mitochondrial loci, but recent studies are moving toward using a comparative genomics approach to address developmental and functional questions. Applying aDNA analysis with more practical outcomes in mind (such as managing conservation) is another increasingly popular trend among studies that utilise avian aDNA, but the majority of these have yet to influence management policy. We find that while there have been advances in extracting aDNA from a variety of avian substrates including eggshell, feathers, and coprolites, there is a bias in the temporal focus; the majority of the ca. 150 studies reviewed here obtained aDNA from late Holocene (100–1000 yBP) material, with few studies investigating Pleistocene-aged material. In addition, we identify and discuss several other issues within the field that require future attention. With more than one quarter of Holocene bird extinctions occurring in the last several hundred years, it is more important than ever to understand the mechanisms driving the evolution and extinction of bird species through the use of aDNA.

## 1. Introduction

As the only living descendants of dinosaurs and with almost ten thousand extant species, the evolution of birds (class: Aves) is a topic that has captured the interest of scientists for hundreds of years. Birds offer excellent cases for the study of evolution and speciation, particularly in island ecosystems—the most famous example being that of Darwin’s Galápagos finches, which were instrumental in the development of his theory of evolution by natural selection. Birds are also of significant cultural and economic importance to people worldwide; their remains have been found in archaeological assemblages throughout history [1]. While birds are the most biodiverse group of terrestrial vertebrates [2], it is estimated that, according to the International Union for Conservation of Nature (IUCN) Red List of Threatened Species, some 147 species of bird have become extinct in recent times, and a further 362 species are currently endangered. Locations such as Hawai’i have lost approximately half of the endemic avifauna that was present before human arrival to these islands 1600 yBP, with an additional 20% disappearing in historic times [3]. Birds are particularly vulnerable to extinction because they are typically large and have highly specialised diet and habitat preferences; thus, birds (particularly flightless birds, of which there are about 60 extant species) are targeted for food, feathers, eggs, etc., and are sensitive to disturbances to their environment [4]. As avian conservation becomes increasingly important (and as the available funding for it becomes increasingly stretched), understanding the mechanisms, both natural and anthropogenic, that have shaped avian evolution and extinction remains key. The analysis of avian ancient DNA (aDNA; defined here as DNA extracted from fossils, artifacts, and sediment, as well as tissue from extinct birds) is one approach that can be used to achieve this. 

**Figure 1:**
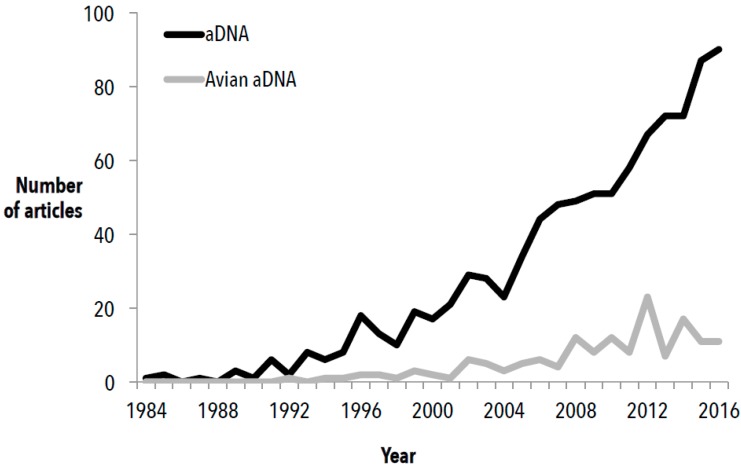
The number of Web of Science articles found containing the terms “(palaeo* DNA) or (paleo* DNA) or (archaeol* DNA) or (archeol* DNA) or (ancient DNA) or aDNA or (extinct* DNA) or (historic* DNA) or palaeogenom* or paleogenom* or archaeogenom* or archeogenom*” in the title (Black), alongside aDNA studies that were ornithological in nature (Table S1) (Grey), each year since the inception of the field (audit accurate as of June 2017). Studies in both categories were manually examined for relevance.

Over the past 30 years, palaeogenetics has grown into a valuable, if not vital, complementary approach to the traditional disciplines of palaeontology, systematics, and phylogenetics for understanding the evolutionary history of both extant and extinct organisms. Ancient DNA can provide direct evidence of the past genetic diversity within species and populations, help assess biodiversity change across time and space to elucidate the causes, and estimate time-of-divergence through molecular dating. While the number of studies utilising aDNA over the past few decades has increased (Figure 1), the proportion of these that examine aDNA from birds is relatively small. Although these figures are likely to be an underestimation of the total number of aDNA studies, they illustrate the trend that, with time, bird aDNA studies represent an increasingly smaller proportion of the total aDNA investigations (Figure 1). 

Here, we review the literature on avian aDNA, highlighting recent advances in the field, and discuss future avenues of research alongside current issues. First, we briefly address the key applications for avian aDNA. Next, we use a meta-analysis of avian aDNA studies to characterise the current landscape of the field, including areas where there is space to expand or initiate research. Finally, we explore the direction in which the field is heading. 

## 2. Avian Ancient DNA Applications

Avian aDNA has been used to investigate diverse topics from phylogenetics to behavior. Table S1 summarises 163 studies that utilise avian ancient DNA in a variety of contexts, some of which are discussed below with reference to the seminal and most recent studies conducted in each area. We focus our attention on studies where DNA has been obtained from Pleistocene and Holocene fossils (both of extant and extinct taxa), or museum specimens of extinct taxa only (note that many studies have been conducted using museum tissue from historic populations of extant species to examine how genetic diversity has changed over time, or for phylogenetics (e.g., [5,6,7,8,9,10]). Some such studies are summarised by Leonard [11], but these have not been included in our meta-analyses). These studies were found by: firstly, using the terms mentioned in the legend of Figure 1 to search the Web of Science database for aDNA studies and manually filtering those that were of an ornithological nature; secondly, manually gathering relevant articles among those referenced by the studies found above, as well as articles that cite the studies found above; and finally, searching NCBI’s GenBank genetic database for sequences of birds that became extinct during the Holocene or Pleistocene and subsequently sourcing the accompanying publication.

### 2.1. Phylogenetics and Biogeography

**Figure 2:**
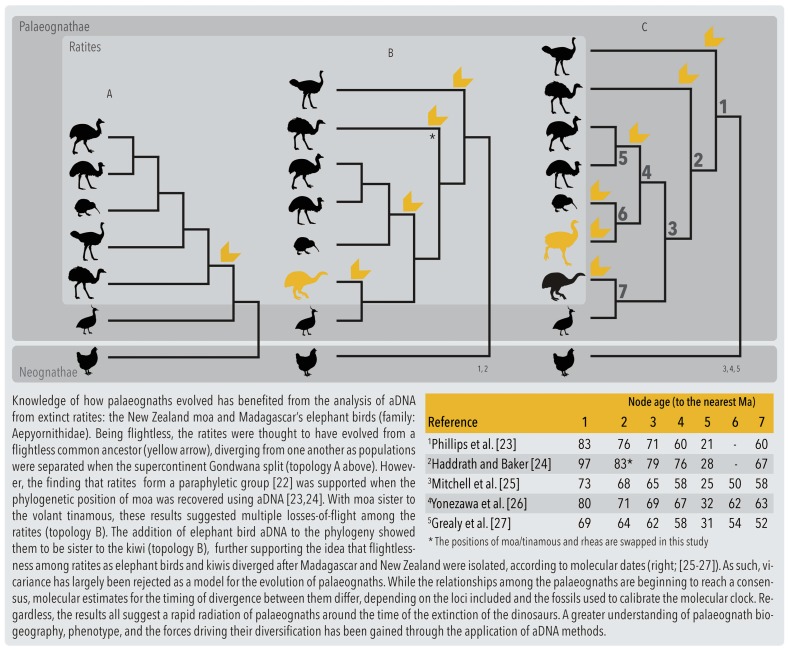
How important is avian aDNA? The palaeognath’s tale. [22,23,24,25,26,27].

The use of aDNA to inform phylogenies has dominated the field since its inception. In 1992, Cooper et al. retrieved the first avian aDNA from moa (Dinornithiformes) tissue specimens, using a 400 base pairs (bp) partial sequence of the mitochondrial *12S ribosomal RNA (rRNA)* gene to reconstruct their phylogenetic relationships with extant ratites. Their results, which showed that the kiwi (*Apteryx* spp.) was more closely related to Australia’s Casuariiformes (emu and cassowary) than its compatriot, the moa, suggested that New Zealand was independently colonised by the ancestors of moa and kiwi [14]. Since then, numerous studies have built upon these results using aDNA from extinct ratites to investigate the origin and evolution of *Palaeognathae* (Table 1, Figure 2) Such studies show that a lack of aDNA potentially limits the reliability of associated evolutionary inferences. Avian aDNA has been instrumental in uncovering the phylogenetic relationships of many extinct birds (Table S1), and is helpful for estimating divergence times using molecular dating (e.g., [15,16,17]), both of which have implications for testing biogeographical hypotheses (e.g., dispersal vs. vicariance; [18]) and evolutionary scenarios (colonisation, speciation; [19,20]). For instance, more accurate rates of evolution can be derived from “time-stamped” aDNA sequences, allowing a better estimation of when lineages diverged, and what events may have driven that divergence [21].

### 2.2. Taxonomy

Ancient DNA has been used to clarify the taxonomy of extinct and extant avian species [28]. For instance, Steeves et al. identified mitochondrial control-region (D-loop) haplotypes in the extinct Tasman booby (*Sula tasmani*) that are identical to a living species (*Sula dactylatra fullagari*), indicating that they are the same taxon, and highlighting how aDNA can complement palaeontology to answer previously intractable questions [29]. In another example, the extinct laughing owl (*Sceloglaux albifacies*) was reassigned to the genus *Ninox*, after aDNA analysis placed it within the *Ninox* clade [30], and similarly, the extinct *Pachyanas chathamica* has been reassigned to the genus *Anas* based on the aDNA analysis (alongside morphological re-analysis) of Holocene fossil bones [31]. For New Zealand’s extinct moa, the aDNA analysis of *Dinornis* bone specimens revealed one sexually dimorphic species of giant moa per island, as opposed to the three previously recognised species described from skeletal morphology [32,33,34]. Understanding taxonomy can be important for identifying evolutionarily significant units that may be of interest for conservation or require alternative management strategies than those currently in place [35].

### 2.3. Domestication

Poultry are an agriculturally and economically important livestock, farmed for meat and eggs. Avian aDNA from poultry can be used to understand domestication, which can provide clues about human behavior. For instance, the domestication of chicken allows us to track human migration and trade patterns by proxy. The mitochondrial control region of ancient chicken specimens from across Polynesia and Southeast Asia revealed the dispersal route of chickens through the Pacific region [36,37] and the world [38]. Genotyping of a ca. 600-yBP chicken bone from Chile provided evidence for possible contact between Polynesians and South Americans prior to European colonisation [39,40]. Ancient DNA from chickens can also reveal the loci involved in their domestication and test hypotheses about how domestic traits were derived: Loog et al., genotyped nuclear genes and found that the frequency of alleles associated with reduced aggression and early-onset egg laying increased in populations around the time chicken consumption intensified, but not during their initial domestication [41].

### 2.4. Palaeoenvironmental Reconstruction

Aspects of the palaeoenvironment can be reconstructed using aDNA derived from environmental samples, such as sediment [42], avian coprolites [43,44,45], and bulk bone [46,47,48]. For example, the amplification of chloroplast aDNA from moa coprolites provided insight into their feeding ecology and niche partitioning [49,50,51], which can have implications for ecosystem rewilding [52,53]; Wood et al. [43] found that the diet of moa does not overlap with the diet of introduced ungulates, refuting the possibility that they may fill the role once played by moa in the ecosystem. In another study, Wood et al. [54] identified a lost ecological connection between two species in serious decline—the kākāpō (*Strigops habroptilus*) and woodrose (*Dactylanthus taylorii*)—using aDNA from coprolites. Their finding that kākāpō fed considerably on woodrose suggests that kākāpō may have been a pollinator of this plant. Using aDNA extracted from sediment, Hofreiter et al. [55] were able to build a picture of past biodiversity in Arizona, amplifying barcoding regions from several taxa, including birds (family: Cathartidae). When coupled with palynological, geological, palaeoclimatic, and isotopic data, the role that different environmental factors played in driving biological change, and the complex relationships between organisms and their environment, can be teased apart (e.g., [56]). On the other hand, aDNA can be used to critically assess the reliability of palaeoecological reconstructions of extinct avian taxa based on these more traditional proxies, such as stable dietary isotopes [57] and the morphological identification of fossils [58].

### 2.5. Zooarchaeology

Human interaction with avifauna can be gauged through the aDNA analysis of artifacts and archaeological middens. DNA extracted from the feathers (*Apteryx* spp.) of Māori cloaks was used to identify the species and provenance through the examination of mitochondrial haplotypes [59]. Such data provided insights into cultural practices including trade, cloak making, and hunting [59]. Oskam et al. [60,61] used DNA extracted from moa eggshell from human burial sites and ovens to show that a large number of eggs from a variety of species were targeted, both for ritualistic purposes and subsistence. In a more recent study, hunting and fishing practices of hunter-gathers in Northern Japan were inferred through the identification of archaeological albatross (*Diomedeidae*) bones using aDNA [62]. In this way, the intensity of prehistoric resource exploitation can be estimated, helping us better understand past human behavior and the anthropogenic factors that contribute to extinction.

### 2.6. Conservation

Knowledge of past genetic diversity, geographic range expansion and contractions, and the biotic and abiotic factors that lead to population decline, fragmentation, and extinction, is critical for making informed conservation decisions, as well as for the sustainable management of certain industries, economies, and local communities. Such knowledge can be revealed through the analysis of aDNA. “Conservation archaeogenomics” or “conservation paleobiology” will play an increasingly important role in conservation in the anthropocene. The mid-1990s saw a shift towards such an approach to aDNA research. For example, Cooper et al. [63] resolved the identity of Holocene fossil bones from Hawai’i by comparing their DNA to that of the endangered Laysan duck (*Anas laysanensis*). Today, the Laysan duck only exists on the remote island of Laysan, north of Hawai’i; however, aDNA analysis of unidentifiable bones showed that these ducks once colonised the main Hawai’ian Islands, but were extirpated upon human colonisation. Cooper et al. [63] used this evidence to justify the reintroduction of the duck to its past range, demonstrating how aDNA could be used for the practical management of vulnerable species. This experiment was the first to highlight how the study of aDNA could have implications for conservation management. 

Recently, aDNA helped track the extinction of *Megadyptes waitaha* and the subsequent recolonisation of their habitat by *Megadyptes antipodes* penguins following human arrival in New Zealand and the Little Ice Age cooling [20,64], highlighting that conservation strategies should take into account the climatic conditions that species are adapted to. In another study, Shepherd and Lambert [65] extracted aDNA from Holocene fossil bones of locally extirpated kiwi species (*Apteryx* spp.) to map the historic distributions of kiwi throughout New Zealand, as well as lost genetic diversity. The restoration and conservation of today’s biodiversity is best achieved with a thorough understanding of how and why it has changed over time. These studies demonstrate how the population history of an extant species, which is directly examinable using aDNA, can be important for informing conservation management strategies.

## 3. The Scope of the Field

Despite the many applications for avian aDNA, we have identified biases in the literature with regard to the types of substrates from which avian aDNA is retrieved, the age of specimens from which aDNA is retrieved, the species and locations studied, the molecular techniques employed, and the genetic loci targeted for sequencing.

### 3.1. Substrates and Species

**Figure 3:**
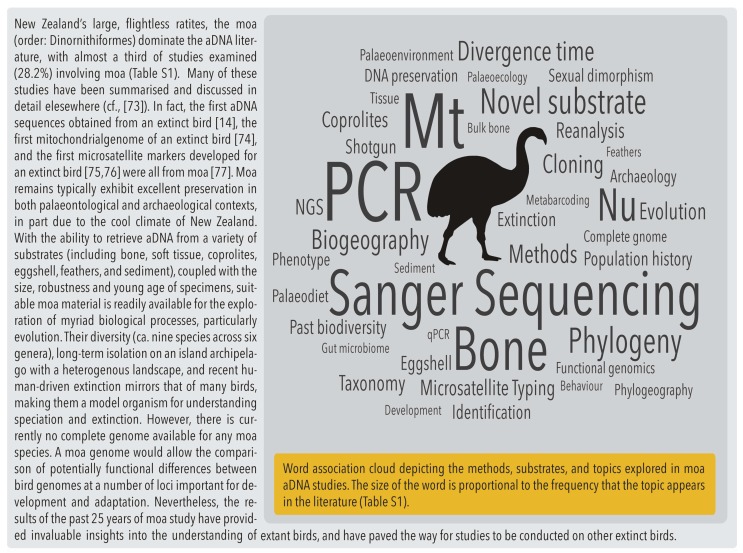
Moa (Dinornithiformes): Extinct model organisms? [14,73,74,75,76,77].

**Figure 4:**
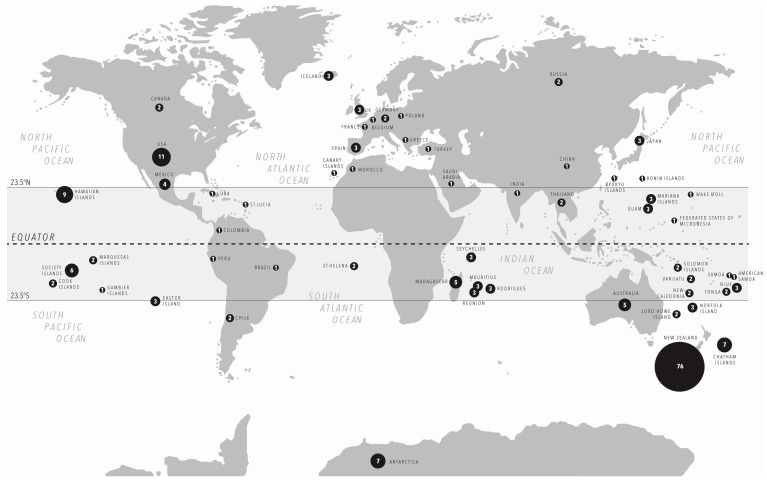
The distribution of the avian ancient DNA studies described in Table S1. The area of the circle is proportional to the number of studies undertaken using samples from that location. The central grey band represents the latitudes that are considered tropical.

For the majority of birds, their ability to fly means that their bodies are small and their bones are hollow; this makes them fragile and less likely to be preserved in the fossil record compared with other animals or flightless birds [66]. In addition, volant species are underrepresented in typical pit-fall deposits because they can fly out [67,68]. As such, most aDNA studies are conducted on large or flightless birds (or both). In fact, over half of the avian aDNA studies conducted in New Zealand are on moa (Figure 3). Large and flightless birds are also more prone to extinction than small, volant birds because, among other things, they have long generation times (and therefore are slower to adapt to selection pressures) [69], are susceptible to predators because they cannot fly away, or suffer from “predator naïvety” [70]. For this reason, the distribution of avian aDNA studies is skewed towards island species (Figure 4). Although island ecosystems offer an opportunity to tease-apart the effects of anthropogenic impacts and climate change, in absolute terms, the diversity of their avifauna is lower than in continental ecosystems: for instance, few studies have been conducted in South America, despite Colombia, Peru, and Brazil being the top-three most bird-rich countries in the world, with over 1800 species each [2]. In contrast, New Zealand has approximately 200 bird species [71], fewer than Australia (with over 800 [72]), yet almost 50% of the avian aDNA studies are on New Zealand species (Figure 4).

These figures are parallel to the over-representation of Holocene-aged specimens (96.3%). Discounting museum specimens, most bone material also hails from the islands of the South Pacific, Indian, and South Atlantic oceans, and represents species that underwent a relatively “recent” extinction. This is most likely because the majority of avian extinctions have occurred since the human occupation of islands in the past several hundred years. In fact, most Pleistocene-aged avian aDNA comes from species that are not extinct but are from cold climates (e.g., 44,000 yBP Adélie penguin bone; [78,79]). Surprisingly, fewer avian aDNA studies have been conducted in the Northern Hemisphere where the cooler climate typically preserves aDNA better than in the Southern Hemisphere (Figure 4).

These biases may be overcome if we use different, perhaps more robust, substrates to retrieve avian aDNA. Advances in extraction protocols have allowed DNA to be recovered from a variety of novel substrates; for example, DNA has been extracted from eggshell [60,80,81,82], feathers [83], sediment [42,55,84], and avian coprolites [43,45,50]. However, of the 156 studies examined that extracted avian aDNA (Table S1), almost half (48.1%) retrieved aDNA from museum skins, with 50% retrieving aDNA from single-source bone, and 13.5% retrieved aDNA from eggshell, coprolites, feathers, sediment, or bulk bone (note that some studies retrieved DNA from more than one source). In many cases, the DNA recovered from these novel substrates displayed a marked improvement in quality and quantity over traditional substrates such as bone and tissue. For instance, Oskam et al. [81] recovered both nuclear and mitochondrial aDNA from moa eggshell, showing that eggshell is conducive to DNA preservation. Furthermore, it was found that DNA retrieved from eggshell has a bacterial load approximately 125 times less than bone of the same age: this may be because eggshell is water resistant and is therefore more protected from microbial decay and hydrolytic damage than bone [81]. Furthermore, Pleistocene-aged aDNA from warm-climate ecosystems has only been retrieved from these “unconventional” substrates [46,48,55].

### 3.2. Molecular Techniques

The advent of next-generation sequencing (NGS) has been instrumental in being able to sequence shorter DNA fragments (i.e., from older or more degraded samples), as well as complex mixtures of aDNA from environmental samples, such as sediment and coprolites. For instance, Murray et al. [46] used an NGS metabarcoding approach to simultaneously identify ten families of Australian birds—including *Phylidonyris* sp. (Australian honeyeater) and *Dasyornis* sp. (bristlebird)—in a Pleistocene fossil assemblage consisting of mixed bone fragments. In the past, such results could only be achieved using the labor-intensive process of cloning polymerase chain reaction (PCR, Table 1) products into a bacterial vector and isolating them again prior to separate Sanger sequencing [45,55]. Furthermore, the high-throughput of NGS allows us to evaluate error and, through shotgun sequencing, assess damage patterns, which serve to authenticate aDNA through characteristic damage profiles. Despite this, there is still a heavy reliance on Sanger (“first-generation” sequencing among avian palaeogeneticists: approximately 78.9% of studies that sequenced avian aDNA utilised a combination of PCR and Sanger sequencing from single-source samples, with only 15.4% employing a NGS approach (either amplicon or shotgun). Even after NGS became widely accessible in the late-00s, since 2010, only 24% of avian aDNA studies have used NGS, with almost 65% still opting for a traditional approach. These figures are somewhat surprising considering the significant advantages that NGS technology brings to the field. However, NGS is becoming more popular, with about 50% of avian aDNA studies utilising NGS in the past two years.

### 3.3. Target Loci

Because aDNA is degraded, studies using it have typically been limited to the analysis of short barcoding regions within mitochondrial genes such as *12S rRNA*, *CO1*, *Cytb*, or the control region/D-loop. This is because mitochondria have a higher copy number per cell than nuclear DNA and thus loci located on this genome have a higher likelihood of being amplified by PCR. However, evolutionary relationships inferred from only one or a few genes in the mitochondrial genome are now considered questionable, as advances in sequencing technology have opened up a new field: palaeogenomics, the study of ancient genomes. It has been possible to sequence complete (or near-complete) mitochondrial genomes of many extinct birds, including moa (Dinornithiformes) [24,74], the elephant bird (Aepyornithidae) [25,26,27], and the Dodo (*Raphus cucullatus*) [16] (Table S1). Mitogenomes provide a larger dataset on which to base phylogenies, and a single run on an Illumina platform generating 15–20 Gb could theoretically sequence over 50 ancient bird mitochondrial genomes with a coverage of 30× at a cost of less than $50 US dollars per genome. (Based on: a 75-cycle single-end mid-output run with an output of 130 million reads, where 85% of those over Q30 are kept; and a mitochondrial genome size of 16,000 bp in a library with 0.5% endogenous mitochondrial sequences that have an average fragment-length of 50 bp.) For instance, Anmarkrud and Lifjeld [85] recovered mitogenomes from 11 extinct bird species in parallel, including the Great Auk (*Pinguinis impennis*), using NGS. High-throughput approaches such as this have the potential to rapidly expand the pool of reference genomes for extinct birds, which is important because many phylogenies are susceptible to taxon sampling bias, where the topology changes depending on which taxa are included in the dataset (Figure 2). Despite this, only around 10% of avian aDNA studies have reconstructed complete mitochondrial genomes from extinct birds. 

However, whole mitochondrial genomes still only represent one genetic locus, and therefore do not necessarily reflect the evolution of avian species [86,87]. Mitochondria evolve approximately 5–10 times faster than nuclear loci [88], and over time, this leads to mutational saturation and rate heterogeneity differences across lineages, making it difficult to accurately resolve deep phylogenetic relationships and estimate divergence times based solely on one locus [89]. Indeed, many phylogenetic relationships and divergence estimates differ depending on the genome from which they are inferred [90]. Accurately determining phylogenetic topology and the timing of divergence is important because, for instance, testing biogeographical hypotheses hinges upon knowing when diversification occurred; as such, many studies opt for a combined approach that integrates both mitochondrial and nuclear loci from protein-coding genes (e.g., [91]), introns (e.g., [92,93]), or transposable elements (e.g., [94]).

Nuclear markers also have a greater resolution to detect population-level changes than mitochondrial markers. For instance, genotyping nuclear microsatellites in individuals offers a rapid method of detecting small differences in genetic diversity between populations, which is essential for fine-scale studies of phylogeography and microevolution [95]. Nuclear microsatellite markers have been developed for extinct organisms, such as the moa [75,76], that were then used to gauge population size and genetic diversity prior to the human colonisation of New Zealand. In addition, the retrieval of nuclear aDNA has allowed unfossilisable phenotypes to be investigated for the first time, as many phenotypes are encoded by nuclear genes. These phenotypes include sexual dimorphism [32], plumage (reconstructed from aDNA extracted from feathers; [83]), and vision [96]. Behaviour can also be inferred from the analysis of nuclear DNA: for example, sex-typing of DNA collected from the surface of moa eggshells suggests that males likely incubated eggs rather than females [80]. Despite the myriad of questions that can be addressed using avian nuclear DNA, only about a quarter of studies have adopted a nuclear perspective, as it remains challenging to obtain.

## 4. Future Research Trajectories

### 4.1. Genomes and Metagenomes

The development of new protocols and techniques over the past decade has made it possible for both mitochondrial and nuclear aDNA to be extracted from older specimens from cool and warm climates. This in turn has allowed a variety of novel and diverse evolutionary questions to be addressed. However, we are now entering what many would consider a “genomics” era, where it is not only feasible but almost expected that some kind of genomic information be used for phylogeny generation, molecular dating, and population histories, even for extinct species. The advent of NGS, combined with the extraction of aDNA from samples with a relatively high endogenous content (such as eggshell) and the use of enrichment methods such as hybridisation capture [97], has allowed thousands of base-pairs of nuclear DNA to be recovered from extinct birds (e.g., [26,27]); however, only one near-complete genome from an extinct bird has been reconstructed thus far. Three near-complete passenger pigeon (*Ectopistes migratorius*) genomes were used to model the past population size through time in order to identify potential drivers of extinction [98]. Complete genomes also allow a detailed analysis of population admixture, dispersal, and selective sweeps. 

Shotgun sequencing ancient samples also generates a wealth of metagenomic data as a by-product. Such data could be used to investigate disease (e.g., pathogens including viruses [99] and parasites), and the gut contents of museum specimens (as well as coprolites) may also contain genetic information about the food consumed, or about the gut microbiome. Wood et al. [100] identified gastrointestinal parasitic worms such as *Cryptosporidium* sp. in moa coprolites using aDNA. This study illustrates how questions regarding parasite-host coevolution and cryptic co-extinctions could be interrogated. However, thus far, analyses of coprolites for examining diet and parasites in birds have not used an NGS approach [44,45,100], despite the method’s potential to recover more taxa, faster. 

If cost and technology are no longer the limiting factors, why aren’t more studies employing a genomic approach, especially when the majority are dealing with specimens less than a few hundred years old? Perhaps the challenge now lies in meeting the immense computational, bioinformatic, and financial demands of analysing genomic data. Access to supercomputing infrastructure is a necessity, but can be expensive and difficult to navigate without an intimate knowledge of programming. Although there are free user-friendly supercomputing resources, data storage, and bioinformatics pipelines available for analysis—including The CIPRES Science Gateway [101], CyVerse [102], and Galaxy [103,104,105])—extremely large datasets require the implementation of software that lacks a graphical interface, which can be limiting for those not fluent in programming languages and scripting. Furthermore, there is also a danger in data mining *post hoc.* In today’s environment, where it is easy to “hoard” data, front-end analysis, including identifying pre-defined hypotheses, is essential—and this is where efforts will need to be concentrated in the future.

### 4.2. Palaeo-Functional Genomics

Another area of focus in the field of aDNA that has barely been touched upon in the avian sphere is palaeo-functional genomics, where the functions and interactions of genes from extinct organisms are compared with their extant relatives. Functional exploration of ancestral versus derived changes in coding regions and non-coding regions can reveal whether such changes were, or are, adaptive. This includes investigating how gene expression is regulated by interactions with other genes, proteins, and the environment. It has been shown that often spatial, temporal, and quantitative changes in gene expression, caused by differences in non-coding regulatory elements [106], microRNAs [107], or epigenetic modifications [108], are responsible for evolution—not necessarily changes in genes themselves [109]. While the function of genes from extinct organisms can be inferred by protein modeling in silico, ancient gene function and regulation has also been examined in vitro. For instance, Huynen et al. [110] sequenced the nuclear *tbx5* gene in moa. This gene is responsible for the development of forelimbs; the authors then expressed this gene in a chicken embryo to determine whether the changes observed in moa *tbx5* play a role in the development of winglessness. It was found that normal forelimb development was induced by moa *tbx5*, indicating that its expression is not likely to account for the loss of forelimbs in moa. In this way, the function of genes from extinct animals can be observed directly. Furthermore, the genomic analysis of extreme forms (such as extinct avian megafauna) facilitates an understanding of the genetic basis of traits such as flightlessness, egg development, beak morphology, and body size. 

Gene expression can also be examined by sequencing RNA or the transcriptome. While RNA is readily retrievable from fresh tissue, its propensity to degrade rapidly makes RNA notoriously unstable and difficult to obtain from ancient samples. However, gene expression is also regulated by epigenetic modifications, such as cytosine methylation. Mapping such modifications in extinct organisms (i.e., the epigenome) can add a new dimension to understanding the complex gene-environment interactions that play a role in evolution. Cytosine methylation patterns can be assessed on a genome-wide level using the bisulphite treatment of DNA combined with NGS [111,112,113,114,115,116]; however, this has yet to be done for any ancient birds.

### 4.3. De-Extinction—A Viable Pursuit?

Being able to express the genes encoded by aDNA may be one step toward resurrecting extinct organisms, a prospect that has always been appealing yet contentious. The controversial idea of “de-extinction” has been circulating for some time, and is led by organisations such as The Genetic Rescue Foundation [117], the Revive and Restore project [118], and the Lazarus project. De-extinction is theoretically achieved by: cloning an extinct organism; by creating a chimeric organism through gene editing that contains some of the genes of an extinct organism in an extant relative; or by reviving or reintroducing lost genetic diversity through the selective breeding of an extant population, or gene editing bottlenecked populations (e.g., CRISPR/Cas-9). Birds constitute a number of candidate species flagged for de-extinction, including the passenger pigeon (*Ectopistes migratorius*), the heath hen (*Tympanuchus cupido*), and moa (Dinornithiformes). Advocates of de-extinction argue that re-establishing an extinct animal to its niche will restore balance to the ecosystem. For instance, small spring annual herbs found in ancient moa coprolites are now critically endangered, potentially because moa played a role in the dispersal of their seeds [43]; bringing back moa may curtail the extinction of such plants.

However, resurrecting extinct birds will be particularly challenging as their complex reproductive system makes cloning a bird unlikely in the near future [119]. In addition, several logistical and ethical issues have been raised against de-extinction [52,120,121,122], not least of which is how to produce a viable, non-inbred population—recent genetic work has shown that cryptic inbreeding can still occur in populations of long lived birds assumed to be secure due to positive population growth [123]. As well as the possibility of re-extinction, de-extinction may stretch already limited conservation funds that could result in further losses of biodiversity, as resources that would be spent resurrecting an extinct species could be used to conserve three-to-eight times as many currently threatened ones [121]. Furthermore, conservation management guidelines do not protect hybrids (e.g., US Endangered Species Act; [124,125]), and are unlikely to protect chimeric de-extinct species. Public opinion toward genetic modification will also influence whether de-extinction technologies will be accepted in avian conservation [126]. Nevertheless, even if de-extinction in the strictest sense is moot, perhaps the attempt will reveal novel strategies to preserve today’s endangered taxa, and lead to a better understanding of gene function and development in birds.

### 4.4. From Ancient DNA to Action

Although many avian aDNA studies have implications for conservation, whether such results translate into conservation policy or have had any applied outcomes is difficult to gauge as there is little communication between policy-makers and the public regarding how science has informed management decisions [127]. For instance, aDNA has helped uncover historic distributions of now extirpated birds, but to our knowledge, this information has only resulted in one instance of a species being reintroduced into their former range: Shepherd et al. [128] used aDNA to establish a pre-human distribution of Haast Tokoeka (*Apteryx australis*), a species of morphologically-cryptic brown kiwi now restricted to an extremely small geographic region. Translocations of Tokoeka into their former range have since been undertaken. However, in the majority of cases, the decision to translocate populations is, at best, based on palaeontological, archaeological, or historical evidence, with no incorporation of aDNA to inform past genetic diversity. The lack of aDNA in these instances can seriously reduce the success of such strategies. 

While the contribution of genetics to conservation management is valued, Taylor et al. [129] have shown that integrating and translating genetics into meaningful conservation management guidelines is problematic due to a lack of funding, access to expertise, understanding of genetics, and communication between geneticists and conservation practitioners. Conservation agencies are also moving away from direct species management to broad-scale ecosystem management, despite avian aDNA in some instances showing that species need individually tailored conservation strategies (e.g., the endangered Otago shag, *Leucocarbo chalconotus*; [10]). Now that a stable foundation has been built between palaeontologists and geneticists, it is time to foster new interdisciplinary collaborations between palaeogeneticists and ecologists, conservation biologists, and governments in order to actively pursue using aDNA technology to inform conservation actions.

## 5. Conclusions

The field of avian ancient DNA has, to date, been heavily focused on Sanger sequencing to select mitochondrial genes from museum specimens of island birds to reconstruct phylogenies, clarify the taxonomy of species, and test biogeographical hypotheses. While this tactic remains robust and valid, avian aDNA is lagging behind the rest of the field, which is rapidly integrating genomic approaches including metagenomics, functional genomics, transcriptomics, and epigenomics. As technology continues to improve exponentially, the field of ancient DNA becomes less about how to obtain sequences, and more about deciphering the function of those sequences, assessing how they have changed, and reconstructing the role that they may have played in evolution across time and space. The challenge that remains now is meeting the immense computational and bioinformatic demands of sequence data; to do this, it will become essential to have well-designed experiments to test a priori hypotheses. Extinct birds are inherently interesting because they are mysterious and unattainable, but sometimes they garner more attention than their extant counterparts. As the list of extinct birds is long and getting longer, we must use what we learn from aDNA to assist in the conservation of today’s endangered birds in a more practical manner.

## Figures and Tables

**Table 1:** Glossary [12,13].

Term	Definition
Palaeognath	A clade of extant birds, sister to Neognaths; retain a “primitive” palate.
Neognath	A clade of extant birds, sister to Palaeognaths; differ from Palaeognathae in the structure of their palate.
Vicariance	The process by which new species are generated through the formation of a geographical barrier to gene flow between populations.
Sexual dimorphism	Disparity in the morphology (typically size) between the males and females of a species.
Coprolite	Fossil faeces.
Palynology	The study of pollen.
Midden	A refuse heap.
Anthropocene	“The period of time during which human activities have had an environmental impact on the Earth regarded as constituting a distinct geological age” [12].
Volant	Possessing the ability to fly.
Predator naïvety	The indifference of island species to potential predators making them vulnerable to predation and extinction.
Next-generation sequencing	NGS; also known as “high-throughput” and “second-generation” sequencing. Short fragments of DNA (typically 50–500 bp) can be sequenced in parallel.
Metabarcoding	Involves the use of highly conserved primers that are able to bind to DNA from multiple different species in a mixed sample, yet amplify a region (a DNA “barcode”) that is variable enough to distinguish between species within the sample based on its sequence [13].
PCR	Polymerase chain reaction; the method by which specific target regions of DNA are amplified.
Sanger sequencing	Also known as “first-generation” sequencing; employs a “chain-termination” chemistry to sequence typically long fragments (400 bp +) with high accuracy, one-at-a-time.
Shotgun sequencing	All DNA fragments within an extract are built into a sequencing library through the ligation of sequences adapters to either end; sequence reads are then overlapped to a continuous sequence. For aDNA, both endogenous and contaminating DNA is sequenced.
Transposable elements	“Jumping genes”; gene sequences that can copy, excise, and reinsert themselves throughout the genome.
Microsatellites	Sequences consisting of short tandem repeats; different alleles are characterised by the number of repeats at a locus.
Hybridisation capture	A method by which to enrich target DNA prior to sequencing through the use of probes from a modern species to “bait” DNA from it’s extinct relative, leaving contaminating DNA behind.
Data mining	“The practice of searching through large amounts of computerised data to find useful patterns or trends” [12].
Front-end analysis	Analysis that occurs prior to the out-set of project in order to plan the most effective way to meet the project’s end-goals.

## Supplementary Materials

The following are available online at www.mdpi.com/2073-4425/8/7/184/s1.
